# Elastic network models for RNA: a comparative assessment with molecular dynamics and SHAPE experiments

**DOI:** 10.1093/nar/gkv708

**Published:** 2015-07-17

**Authors:** Giovanni Pinamonti, Sandro Bottaro, Cristian Micheletti, Giovanni Bussi

**Affiliations:** Scuola Internazionale Superiore di Studi Avanzati, International School for Advanced Studies, 265, Via Bonomea I-34136 Trieste, Italy

## Abstract

Elastic network models (ENMs) are valuable and efficient tools for characterizing the collective internal dynamics of proteins based on the knowledge of their native structures. The increasing evidence that the biological functionality of RNAs is often linked to their innate internal motions poses the question of whether ENM approaches can be successfully extended to this class of biomolecules. This issue is tackled here by considering various families of elastic networks of increasing complexity applied to a representative set of RNAs. The fluctuations predicted by the alternative ENMs are stringently validated by comparison against extensive molecular dynamics simulations and SHAPE experiments. We find that simulations and experimental data are systematically best reproduced by either an all-atom or a three-beads-per-nucleotide representation (sugar-base-phosphate), with the latter arguably providing the best balance of accuracy and computational complexity.

## INTRODUCTION

Characterizing the functional dynamics of RNA molecules is one of the key standing issues in molecular biology. The interest in this topic is spurred by the ongoing discovery of ever new biological roles that RNAs can have in different contexts (see, e.g. (1) for a recent review) and, at the same time, by the realization that the structure → function relationship of these molecules is often related to their internal dynamics (2). In this respect, theoretical approaches hold much potential for complementing experiments and provide valuable quantitative insight into the functional dynamics of RNAs. For instance, molecular dynamics (MD) simulations with atomistic force fields have been used to reproduce experimental measurements and aid their interpretation (see, e.g. (3–9)). However, it may be argued that one of the most important limitations to the systematic use of atomistic MD simulations for characterizing the behavior of RNA is their intensive computational demand. In fact, most if not all current MD studies are still limited to the µs timescale.

For this reason, several efforts are being spent toward developing coarse-grained approaches capable of striking a good balance between accuracy and computational efficiency (see, e.g. (10–17)). In this respect, it should be noted that coarse-grained models are valuable not only because they are amenable to extensive numerical characterization, but precisely because their simplified formulation can offer important insight into the main physico-chemical mechanisms that underpin the behavior and properties of a given biomolecule.

For proteins, a successful class of such simplified models are elastic networks. These models were originally motivated by the seminal work of Tirion (18) who showed that the Hessian of the potential energy of a globular protein computed from an atomistic force field could be reliably reproduced by replacing the detailed inter-atomic forces by spring-like, harmonic interactions. This remarkable fact was rationalized *a posteriori* in terms of the large-scale character that low-energy fluctuations have in proteins, which makes them amenable to be captured with models that are oblivious of the details of the potential (19–23). This observation, in turn, prompted further development of simplified harmonic models where the structural descriptions themselves were simplified by reducing the number of interaction centers, also termed beads. In their simplest formulation, elastic network models (ENMs) incorporate harmonic interactions between pairs of C_α_ beads (19,21–22,24) while two-beads amino acid representations, e.g. for the main- and side-chains (22), can predict structural fluctuations in very good accord with atomistic MD simulations (25).

By comparison with proteins, the development and application of elastic networks aimed at nucleic acids is still relatively unexplored. Bahar and Jernigan first applied network models to the conformational dynamics of a transfer RNA using a model with two beads per nucleotide (26). Several authors further simplified this model using a single bead placed on the phosphorus atom (27–33). More recently, Setny and Zacharias suggested that the best candidate to host a single ENM bead is the center of the ribose sugar in the backbone (34). Other ENMs with more beads per nucleotide have also been used (24,28,33,35). Most of these studies assessed the validity of different representations by focusing on their capability to reproduce either the structural variability observed across experimental conformers or the Debye–Waller factors from X-ray experiments. ENM fluctuations were also compared with accurate atomistic MD simulations, but the comparison was either limited to short timescales (29) or to model simple double helices (34).

Toward the goal of identifying the most suitable RNA ENM, here we assess the performance of an extensive repertoire of ENMs which are all equally viable *a priori*. These models, in fact, differ for the specific single- or multi-bead representations used for each nucleotide, as well as for the spatial range of the pairwise elastic interactions. As stringent term of reference we perform µs timescale atomistic MD simulations on RNA molecules containing canonical A-form double helices as well as nontrivial secondary and tertiary structures. Additionally, we introduce a procedure to compare fluctuations with selective 2′-hydroxyl acylation analyzed by primer extension (SHAPE) experiments (36,37). SHAPE reactivity is empirically known to correlate with base dynamics and sugar pucker flexibility at the nucleotide level (38) and hence is, in principle, well suited for validating predictions of RNA internal dynamics. Recently, Kirmizialtin *et al*. have proposed a link between fluctuations of selected torsional angles and SHAPE reactivity and used SHAPE data as an input to improve the accuracy of force-field terms in an atomistic structure-based (Go-like) model (39). However, to the best of our knowledge, the present study is the first attempt of using SHAPE reactivity measurements to assess the predictive accuracy of three-dimensional coarse-grained models or atomistic MD simulations.

We find that the best balance between keeping the model complexity to a minimum and yet have an accurate description of RNAs’ internal dynamics is achieved when each nucleotide is described by three beads representing the sugar, the base and the phosphate (SBP) groups. Slightly better results can be obtained using the much more computationally demanding all-atom (AA) model. As a matter of fact, the SBP and AA ENMs can reproduce to a very good accuracy the principal structural fluctuations as predicted from µs-long atomistic MD simulations, both in their directions and relative amplitudes. Additionally, they provide a satisfactory proxy for the nucleotide-level flexibility as captured by experimental SHAPE data.

## MATERIALS AND METHODS

### RNA dataset

**Figure 1.**
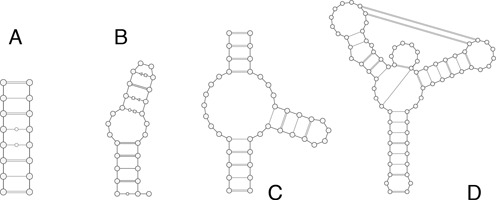
Secondary structures of the four molecules studied: **A**: eight-base-pairs duplex; **B**: sarcin-ricin domain; **C**: hammerhead ribozyme; **D**: *add* adenine riboswitch.

We performed atomistic MD simulations on four different RNA molecules (Figure 1). These systems were chosen so as to cover a variety of size and structural complexity and yet be amenable to extensive simulations, as detailed in Table 1.

**Table 1.** RNA dataset: details and length of MD simulations

System	PDB code	Chain length	Simulation time (µs)
Duplex	1EKA	16	1.0
Sarcin-ricin domain	1Q9A	25	0.9
Hammerhead ribozyme	301D	41	0.25
*add* riboswitch	1Y26	71	0.25
*thiMr*iboswitch	2GDI	78	N.A.

For the thiM riboswitch, no MD was performed.

The first entry is the NMR-derived model of the $^{\tt {GAGUGCUC}}_{\tt {CUCGUGAG}}$ RNA duplex, featuring two central G-U Wobble pairs (40). As a second system, we considered the sarcin-ricin domain (SRD) from *Escherichia coli* 23S rRNA, which consists of a GAGA tetraloop, a flexible region with a G-bulge and a duplex region (41). The U nucleobase at the 5′ terminal was excised from the high-resolution crystal structure. We further considered two more complex molecules: the hammerhead ribozyme (42) and the *add* adenine riboswitch (43). Both systems are composed of three stems linked by a three-way junction. In the *add* riboswitch, two hairpins are joined by a kissing loop interaction. All these systems, except for the duplex, were previously characterized by various computational means, including atomistic MD simulations (29,44–49).

### MD simulations

All MD simulations were performed using GROMACS 4.6.7 (50) with the AMBER99 force field (51) including parmbsc0 (52) and χ_*OL*3_ (53) corrections. GROMACS parameters can be found at http://github.com/srnas/ff. The trajectories were obtained in the isothermal-isobaric ensemble (*T* = 300 K, *P* = 1 atm) with stochastic velocity rescaling (54) and Berendsen barostat (55). Long range electrostatics were treated using particle-mesh-Ewald summation (56). The equations of motion were integrated with a 2 fs time step. All bond lengths were constrained using the LINCS algorithm (57). Na^+^ ions were added in the box in order to neutralize the charge, and additional Cl^−^ and Na^+^ at a concentration of 0.1 M. AMBER-adapted parameters were used for Na^+^ (58) and Cl^−^ (59). The adenine ligand bound to the *add* riboswitch was parametrized using the general Amber force field (gaff) (60) and partial charges were assigned as discussed in reference (48). The analyses of the hammerhead ribozyme and of the *add* riboswitch trajectories were performed after discarding the first 10 ns and 5 ns, respectively.

### Elastic Networks

In ENMs a simplified structural representation is achieved by representing any monomeric unit of the biopolymer with one or more beads. Accordingly, the model potential energy is equivalent to the one of a set of *N* beads connected by pairwise harmonic springs which penalize deviations of inter-bead distances from their typical, reference values. Thus, the elastic network does not directly restrain the absolute positions of the beads but only their distances. In the simplest formulation, the spring constant of the harmonic pairwise interaction is set equal to a master spring constant *k* whenever the reference distance between the two beads is smaller than a pre-assigned interaction cutoff (*R*_*c*_), and set to zero otherwise.

The potential energy of the system can be approximated to second order as

(1) \begin{equation*} U(\delta r_{i,\mu } , \delta r_{j,\nu }) \approx {1 \over 2} \delta r_{i,\mu } M_{i j, \mu \nu } \delta r_{j,\nu } \end{equation*}

where the 3*N* × 3*N* symmetric matrix, *M*, is the Hessian of *U*, and δ*r*_*i*, μ_ is the μ Cartesian component of the deviation of bead *i* from its position in the reference structure.

#### Repertoire of possible elastic networks for RNAs

In protein contexts, the standard formulation of ENMs is based on the intuitive amino acid representation with primary interaction centers located on the mainchain (e.g. the C_α_ atoms) and possibly auxiliary ones for the sidechains (22). By analogy with the case of proteins, one may expect that the primary ENM interaction centers could be the phosphate groups, which provide the backbone connectivity for single RNA strands (27–33). Besides this possibility, we here investigated alternative representations considering all possible ENM combinations based on the use of one or more interaction centers representing the three chemical groups of each nucleotide: the SBP (in short S, B and P, respectively). Each group is represented by a specific atom, namely C1′ for the sugar, C2 for the base and P for the phosphate group. This selection follows from the customary coarse-graining choices previously adopted in various contexts (10), including elastic networks (24,33–35). For each model the interaction cutoff distance, *R*_*c*_, is varied in the 3–30 Å range with 1 Å increments so as to assess the dependence of the predictions on the degree of connectivity of the elastic network.

#### Reference structure

For each RNA dataset entry, the reference structure for ENM calculations is set equal to the centroid structure of the associated MD trajectory. This is the conformer with the lowest average mean square distance from all MD-sampled structures after an optimal rigid structural alignment (61). In the case of the *add* riboswitch, the adenine ligand atoms are included in the ENM calculation.

### Comparison of ENMs and MD

For a detailed and stringent comparison of ENM and MD we shall consider the covariance matrix, which provides information on the structural fluctuations at equilibrium. The MD covariance matrix entries are defined as $C^{\text{MD}}_{i j, \mu \nu } = \langle \delta r_{i,\mu } \delta r_{j,\nu } \rangle \;, \; \; \textrm {with} \quad \delta r_{i,\mu } = (r_{i,\mu } - \langle r_{i,\mu }\rangle )$ where *i* and *j* run over the *N* indexed interaction centers, *μ* and *ν* run over the Cartesian components and 〈〉 denotes the time average over the sampled conformations after an optimal structural superposition over the reference structure. When comparing with a coarse-grained ENM, the structural alignment and the calculation of *C*^MD^ are both performed by exclusively considering the same atom types used as beads in the ENM. For ENM, the covariance matrix is obtained from the pseudoinverse $\tilde{M}^{-1}$ of the interaction matrix defined in Equation (1), as $C^{\text{ENM}}_{i j, \mu \nu } = k_B T \tilde{M}^{-1}_{i j, \mu \nu }$. Here *k*_*B*_ is the Boltzmann constant and *T* is the temperature. We observe that the *k*_*B*_*T* term is here required to allow the absolute covariance matrix to be properly related to the spring stiffness *k*. However, since in all the comparisons discussed below we always consider a multiplicative term in the covariance matrix as a parameter for the fitting procedures, the values of both *k*_*B*_*T* and *k* are never used in practice.

#### Effective Interaction Matrix

When comparing different ENMs one must consider only the modes related to the fluctuations of the degrees of freedom in common between the models. To achieve this, it is necessary to separate the degrees of freedom of the beads of interest (with subscript *a* in the following) from the others (with subscript *b* in the following) and compute the effective interaction matrix of the former (23,62–64). This is accomplished by formally recasting the interaction in the following block form $M= \left( \begin{array}{c|c}M_a & W \\ \hline W^T & M_b \\ \end{array} \right)$ where *M*_*a*_ and *M*_*b*_ are the interaction matrices of the two subsystems, while *W* represents the interactions between them. The effective interaction matrix governing the dynamics of subsystem *a* alone is

(2) \begin{equation*} M^{\textrm {eff}}_a = M_a - W M_b^{-1} W^T \end{equation*}

For a detailed derivation of this equation see (63). Using this effective matrix one can compute the fluctuations relative to the subsystem considered.

#### Measures of similarity between essential spaces

The comparison of the essential dynamical spaces of ENM and MD simulations is here carried out by considering two quantities, namely the Pearson correlation of mean square fluctuation (MSF) profiles and the similarity between the eigenspaces of covariance matrices.

The MSF of a given center, *i*, can be obtained in the MD simulation by time-averaging the mean square displacements. Similarly, in ENMs they are given by $\text{MSF}_i = \langle \delta r_i^2 \rangle = k_B T \sum _{\mu =1}^3 \tilde{M}^{-1}_{ii,\mu \mu }$. We remark that the amplitudes of fluctuations are known to be inversely correlated to the local density, that is the number of neighboring centers (65). We also recall that the MSF profile is computed after carrying out an optimal global structural superposition of all sampled conformers. As a consequence, the MSF of any given center depends not only on the local structural fluctuations but on the global intra-molecular ones too.

The accord of two covariance matrices, *A* and *B*, can be measured more directly by comparing their essential dynamical spaces, identified by the set of their eigenvectors $\lbrace \mathbf {v}_A\rbrace$ and $\lbrace \mathbf {v}_B\rbrace$ and eigenvalues {λ_*A*_}, {λ_*B*_}. A stringent measure of this consistency is the root weighted square inner product (RWSIP) (66)

(3) \begin{equation*} \text{RWSIP}= \sqrt{\frac{\sum _{i,j=1}^{3N} \lambda _{A,i} \lambda _{B,j} (\mathbf {v}^i_A \cdot \mathbf {v}^j_B )^2 }{\sum _{i=1}^{3N} \lambda _{A,i} \lambda _{B,i}} } \end{equation*}

which takes on values ranging between 1, when the two ranked dynamical spaces coincide, and 0, when they are completely orthogonal.

The statistical significance of both the MSF correlation and the RWSIP is assessed by using two terms of reference. The first one is given by the degree of consistency of the MSF or RWSIP for first and second halves of the atomistic MD trajectories. This sets, in practice, an upper limit for very significant correlations of the observables. The second one is the degree of consistency of the random elastic network of Setny *et al*. (34) with the reference MD simulations. This is a fully connected elastic network where all pairs of beads interact harmonically though, for each pair, the spring constant is randomly picked from the [0, 1] uniform distribution. Because this null ENM does not encode properties of the target molecule in any meaningful way, it provides a practical lower bound for significant correlations between ENMs and MD simulations.

### Comparison with SHAPE data

To compare the fluctuations from both ENMs and MD simulations with data from SHAPE experiments we here scrutinize several order parameters that, *a priori* could be viable proxies for SHAPE reactivity data, namely: i) the variance of the distance between selected pairs of beads and ii) the variance of the angle between selected triplets of beads. The variance of each distance and angle as obtained from MD was compared with the SHAPE reactivity of the corresponding nucleotide for the *add* riboswitch taken from (67). Distances and angles were computed using PLUMED (68).

In the ENM framework, the variance of the distance between two beads can be directly obtained from the covariance matrix in the linear perturbation regime as

(4) \begin{equation*} \sigma _{d_{i j}}^2 = \sum _{\mu ,\nu =1}^{3} \frac{ \tilde{d}_{i j}^{\mu } \tilde{d}_{i j}^{\nu }}{\tilde{d}^2} ( C_{i i,\mu \nu } + C_{j j,\mu \nu } - C_{i j,\mu \nu } - C_{j i,\mu \nu }) \end{equation*}

where $\tilde{d}_{i j}^{\mu }$ is the μ Cartesian component of the reference distance between bead *i* and *j*.

When comparing ENM and SHAPE we also considered the experimental data relative to the *thiM* thiamine pyrophosphate riboswitch published in (67). For this molecule no reference MD simulation was performed and ENMs were computed directly on the crystal structure (PDB code: 2GDI) (69).

## RESULTS

**Figure 2.**
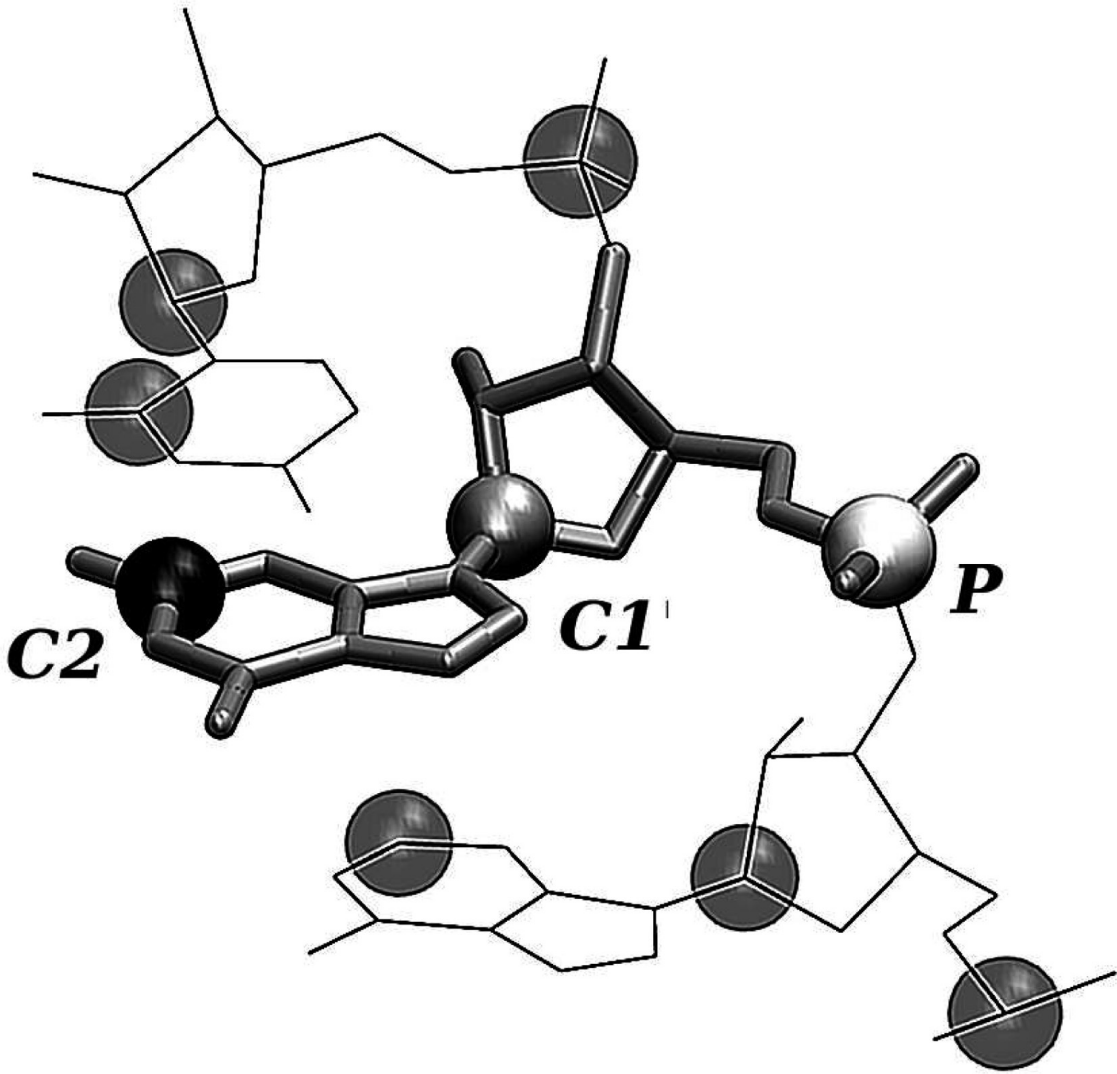
Schematic representation of the beads used to construct the ENM. The three atoms used as beads are the C2 carbon in the base, the C1′ carbon in the sugar ring and the P atom in the phosphate group, as indicated by labels.

For the comparative validation against MD and SHAPE data we consider eight different types of elastic networks, as summarized in Table 2. A subset of the considered models have been previously used in different contexts (24,33–35). With the exception of the AA model, all other ENMs will be referred to with the one-, two- and three-letter acronyms corresponding to which of the phosphate (P), sugar (S) or base (B) interaction centers are used, see Figure 2. We also tested ENMs with a higher number of beads (see Supplementary Figure S1 for an example). All the considered ENMs feature a sharp-cutoff interaction scheme (as explained in the Materials and Methods section). Using a distance-dependent elastic constant yields similar results (Supplementary Figure S2 for details).

**Table 2.** Summary of the tested ENMs

ENM	C1′	C2	P	Others	Best *R*_*c*_ (Å)	Number of neighbors
P			✓		20	15.3
S	✓				15	9.9
B		✓			17	14.8
SP	✓		✓		19	30.4
BP		✓	✓		18	29.9
SB	✓	✓			11	15.4
SBP	✓	✓	✓		9	12.0
AA	✓	✓	✓	✓	7	52.9

For each model, the adopted beads are marked. AA include all heavy atoms. Values of the cutoff radius (*R_c_*) that maximize the RWSIP and average number of neighbors are also shown.

### Comparison of ENMs and MD

**Figure 3.**
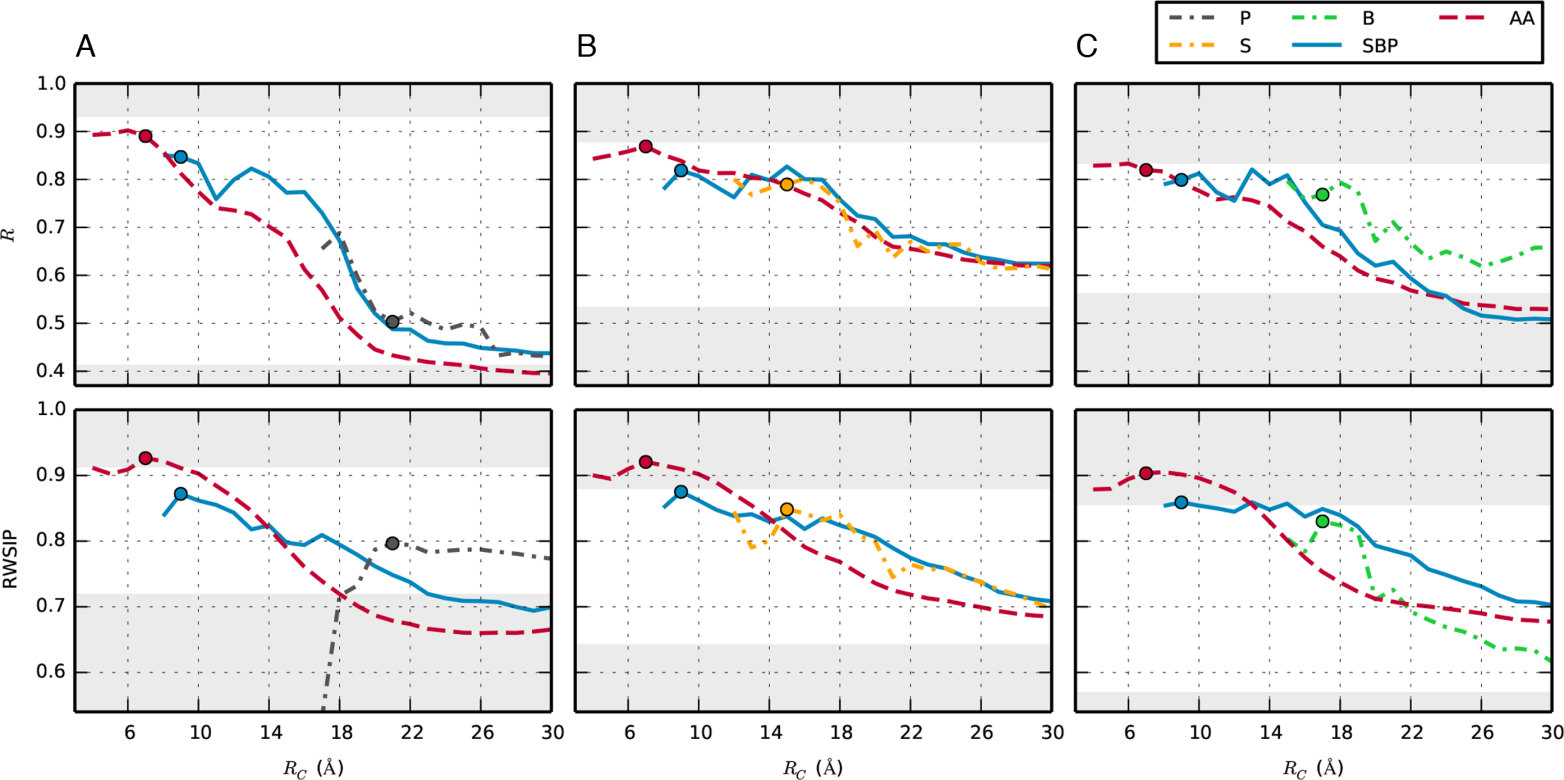
Agreement between MD simulations and ENM for different radii of cutoff. Correlation between MSF (upper panels) and RWSIP (lower panels). Values at the optimal cutoff values are represented by circles. **A**: phosphate beads; **B**: sugar beads; **C**: nucleobase beads. The gray regions correspond to values below the random-network model or above the MD self-agreement.

The consistency of ENM and MD simulations was assessed by computing the Pearson correlation coefficient (*R*) for the MSF profiles and the RWSIP for the essential dynamical spaces. To keep the comparison as simple and transparent as possible, each measure was computed separately for the S, B and P interaction centers. For multi-center ENMs this required the calculation of the effective interaction matrix (Equation (2)). Using as a reference the experimental structure in place of the MD centroid introduces only minor differences in the results, see Supplementary Figure S3. Each measure was then averaged over the four systems in Table 1 (see Supplementary Figure S4 for non-averaged values). The results, shown in Figure 3, are profiled as a function of the elastic network interaction cutoff distance, *R*_*c*_. The smallest physically viable value for *R*_*c*_, that is the abscissa of the left-most point of the curves, is the minimum value ensuring that the ENM zero-energy modes exclusively correspond to the six roto-translational modes.

The main feature emerging from Figure 3 is that, across the various models, the highest consistency with MD is attained when *R*_*c*_ is marginally larger than its smallest physically viable value. It is also noted that the minimum value of *R*_*c*_ varies significantly across the models: for the AA model, which is the most detailed ENM, it is as low as 4 Å, while for the single-bead ones it is often larger than 10 Å. The MSF and RWSIP accord both decrease systematically as *R*_*c*_ is increased starting at the optimal value. This fact, which to our knowledge has not been reported before, can be rationalized *a posteriori* by considering that upon increasing *R*_*c*_, one endows the network with harmonic couplings among nucleotides that are too far apart to be in direct physical interaction, and this brings about a degradation in model performance.

Furthermore, it is noted that the detailed, but also computationally more onerous, AA model is consistently in better accord with MD data than any of the coarse-grained ENMs. For this model, the degree of ENM–MD consistency is practically as high as the internal MD consistency at the optimal value *R*_*c*_ ≈ 7 Å, or even higher in some cases. As a general trend, we notice that the accord between MD and ENMs decreases for coarser models (see also Supplementary Figure S5 for models including two beads per nucleotide). Importantly, the AA and SBP models perform well not only on average but for each considered structure, whereas the performance of models with fewer interactions centers is less consistent across the repertoire of RNA molecules, see Supplementary Figure S4. For all models, considering the optimal value of *R*_*c*_ both MSF and RWSIP accord are significantly higher than for the null model, indicating that all the ENMs are overall capable to capture the salient physical interactions of the system.

It is important to mention here that in the MD simulation of the duplex we observed a fraying event at time ≈670 ns (see Supplementary Figure S6), followed by a re-zipping into the native structure. As a matter of fact, fraying events are expected at RNA termini on the µs timescale covered by our simulations (70). In spite of the fact that these events are clearly out of the linear perturbation regime where one would expect ENM to properly predict fluctuations, the correlation between MD and ENM is reasonably high. By removing from the analysis the highly fluctuating terminal base pairs, the correlation is further improved (Supplementary Figure S7).

In Table 2 we summarize all the results for the optimal cutoff radius, determined as the radius that maximizes the RWSIP. The last column of the table reports the average number of neighbors of a bead, that is the number of other beads at distance smaller than *R*_*c*_ from it.

#### Effect of ionic strength

One standing question for RNAs, that is relevant also for ENM development (33), is whether and how the internal dynamics of these biomolecules is affected by the concentration and type of counterions in solutions. These parameters, in fact, modulate the screening of the electrostatic self-repulsion of RNA backbone and are indeed often used to artificially induce RNA unfolding. Because current formulations of ENMs, including those considered here, do not explicitly account for electrostatic effects, and thus intrinsically provide results that are independent of the ionic strength, it is important to ascertain to what extent changes of ionic strength would affect the collective internal dynamics of the considered RNAs.

To clarify this point, we carried out MD simulations at different nominal concentrations of monovalent salt Na^+^/Cl^−^. The consistency of the essential dynamical spaces observed in simulations based on different salt concentrations was measured with the RWSIP. Only the C2, C1′ and P atoms were considered for computing the essential dynamical spaces.

As summarized in Table 3, the essential dynamical spaces are very consistently preserved over a wide range of ionic strengths. This finding complements a recent study of Virtanent *et al*. (71) where the electrostatic free energy was shown to be minimally affected by ionic strength. In the present context, the result justifies the use of RNA elastic networks with no explicitly treatment of the ionic strength. It is however important to note that our test was limited to monovalent cations. The treatment of divalent cations is known to be very challenging because of force-field limitations and sampling difficulties.

**Table 3.** RWSIP between 100 ns trajectories at different NaCl concentrations and the 500 ns trajectories at 0.1 M

Molecule	0.0 M	0.1 M	0.5 M	1.0 M
Duplex	0.938	0.998	0.991	0.990
SRD	0.983	0.983	0.982	0.993

For the duplex, only the first half of the 1 µs trajectory was considered, thus discarding the contribution of the base fraying event (see Supplementary Figure S6).

We finally notice that in our simulations with standard AMBER ions we did not observe any ion-crystallization event (72). For maximum robustness we tested the alternative ion parameterization by Joung and Cheatham (73), obtaining very similar results.

### Comparison with SHAPE data

To complement the validation of ENM against MD, we assessed their consistency with experimental data too. To this purpose we considered data obtained from SHAPE experiments, which probe RNA structural fluctuations at the nucleotide level (38). One standing challenge is that it is not yet settled which simple structural or dynamical observables can be used as viable proxies for the SHAPE intensities. To tackle this elusive problem, we first set out to analyze the MD simulations so as to identify the local fluctuations that best correlate with SHAPE data. Specifically, we compared our MD simulation and available SHAPE data for the *add* riboswitch (67). A related comparison based on B-factor profiles, which are commonly used to validate ENM predictions (albeit with known limitations (25)) is provided in Supplementary Figure S8.

**Figure 4.**
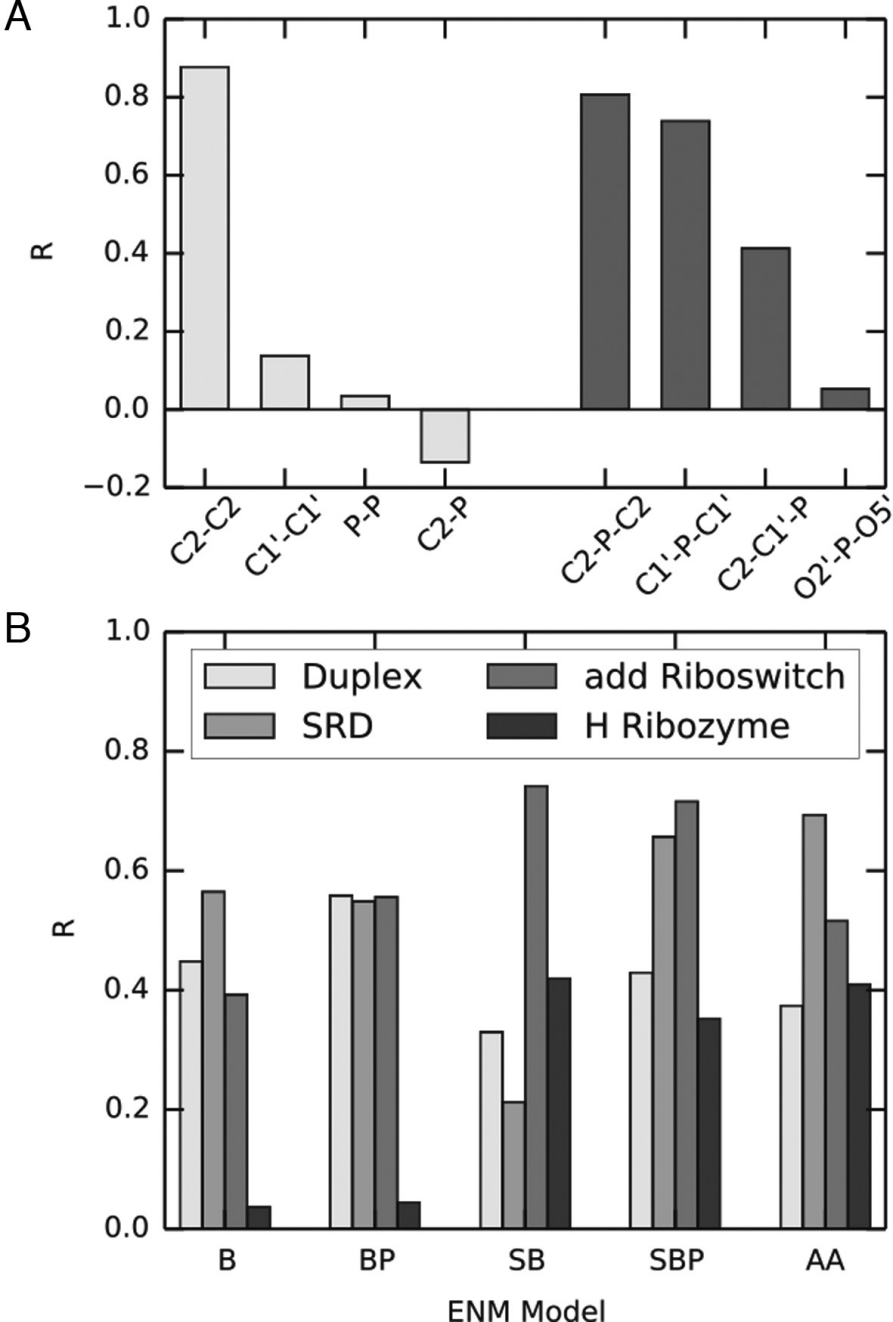
**A**: Pearson correlation coefficient *R*, computed between SHAPE reactivities and the fluctuations of different distances (light grey), and angles (dark grey), computed from the MD trajectory of the *add* riboswitch. Residue indexes are shown in Supplementary Figure S10; **B**: correlation between the fluctuations of the distance of consecutive C2 atoms, from the MD simulation and from the different ENMs.

**Figure 5.**
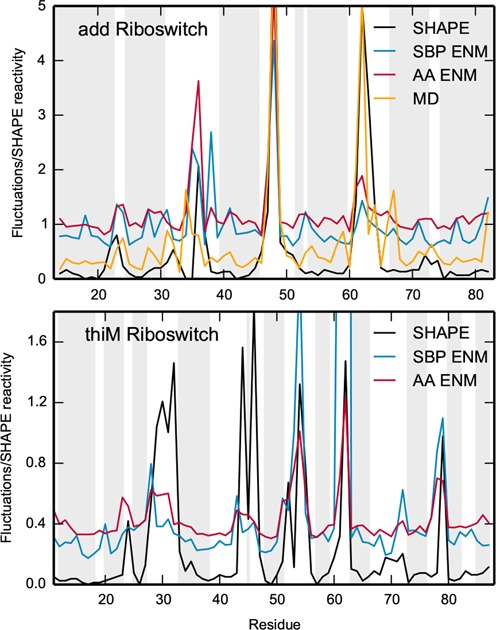
Comparison of the flexibility of the *add* riboswitch (upper panel) and the *thiM* riboswitch (lower panel). The SHAPE reactivities (black) are compared with the C2-C2 fluctuations predicted by the SBP and the AA models. For the *add* riboswitch, also fluctuations from MD are shown. Regions corresponding to residues forming Watson–Crick or wobble base pairings are shown in gray.

As it emerges from Figure 4A, the best correlation with experimental SHAPE reactivity was found for the fluctuations of the distance between consecutive C2 atoms (*R* = 0.88). This is remarkable, since the SHAPE reaction does not explicitly involve the nucleobases. These fluctuations are shown, as a function of the residue index, in Figure 5. The result can be interpreted by considering that most of the structural constraints in RNA originate from base–base interactions, and fluctuations in base–base distance are required for backbone flexibility. The fluctuations of the angle O2′-P-O5′ instead showed a poor correlation with experimental SHAPE data (*R* = 0.05). We notice here that the value of this angle has been shown to correlate with RNA stability related to in-line attack (74), and its fluctuations were recently used in the SHAPE-FIT approach to optimize the parameters of a structure-based force field using experimental SHAPE reactivities (39). We also observe that the fluctuations of the distance between consecutive C2 atoms could be correlated with ribose mobility, which in turn depends on sugar pucker (75,76). Interestingly, C2′-endo conformations have been shown to be overrepresented among highly reactive residues in the ribosome (38). A histogram of C2-C2 distances for selected sugar puckers is shown in Supplementary Figure S9, indicating that C2′-endo conformations correspond to a larger variability of the C2-C2 distance. In conclusion, although the scope of the present SHAPE profiles comparison could be affected by the limited accuracy or precision of both experimental and MD-generated data, the obtained results suggest that a good structural determinant for SHAPE reactivity is arguably provided by base–base distance fluctuations. In Supplementary Figure S10 we show this comparison using a non-parametric measure of correlation.

Based on this result, we next quantified to which extent the ENMs are able to reproduce the profile of fluctuations of the C2-C2 distance. This test complements the assessment made using MSF and RWSIP, which mostly depends on the agreement of large-scale motions and does not imply a good performance in the prediction of local fluctuations. This comparison is presented in Figure 4B where the ENM–MD Pearson correlation coefficients for each considered ENM are summarized.

We remark here that the duplex (1EKA) is undergoing a base fraying, so that MD exhibits very large fluctuations at one terminus (see Supplementary Figure S6). The overall accord between MD and ENM is moderately good, although significantly worse than the accord with the large-scale motions presented before. Overall, it is seen that the both the SBP model and the AA models provide the best agreement.

In the following, we thus test whether the SBP and AA models are capable of reproducing SHAPE reactivities directly, without the need for an expensive MD simulation to be performed. ENM and SHAPE data were compared for two different molecules, namely the aforementioned *add* riboswitch and the *thiM* riboswitch.

As we can see from Figure 5 the predictions of ENM are in qualitative agreement with the SHAPE data. In particular, high SHAPE reactivity in the loop and junction regions correspond to highly fluctuating beads, both for the *add* and *thiM* riboswitch. We notice that this agreement goes beyond the mere identification of the residues involved in Watson–Crick or wobble pairings (77), as there appear several unpaired bases with a low SHAPE reactivity. This feature seems to be often correctly reproduced by the C2-C2 fluctuations profile. By visual inspection, it can be seen that non-reactive, non-paired bases often engage non-Watson–Crick base pairs as well as stacking interactions, as shown in Supplementary Figure S11. The Pearson correlation coefficients are summarized in Table 4. In this case too, it is found that the AA ENM performs better than the SBP ENM which, nevertheless, is much less demanding computationally because of its simpler formulation.

**Table 4.** Pearson correlation coefficients between C2-C2 fluctuations predicted by ENM/MD and SHAPE reactivities

Molecule	SBP	AA	MD
*add*	0.64	0.76	0.88
*thiM*	0.37	0.59	N.A.

## DISCUSSION

The development and performance assessment of elastic networks for RNAs have so far been pursued in two main directions. On one hand, Zimmermann and Jernigan (33) have recently shown that the essential dynamical spaces of ENMs based on the phosphate representation of RNAs can satisfactorily account for the structural variability observed across crystal structures homologs. On the other hand, Setny and Zacharias (34) have considered ENMs where different atoms of the RNA backbone (i.e. sugar and phosphate groups only) are alternatively used to represent nucleotides in short RNA duplexes. Within this class of single-bead ENMs and target RNA structures, it was found that those based on the sugar-group representation yielded the structural fluctuations with the best consistency with MD simulations or nuclear magnetic resonance ensembles (34).

Here, we tackle this standing challenge by searching for the simplest and yet accurate RNA ENM. We analyze a comprehensive combinations of (i) interaction centers, or beads, for each nucleotide and (ii) spatial range of the elastic interaction. In total, we considered eight different types of ENMs, which are listed in Table 2. For the critical assessment of their performance, we validated the predicted structural fluctuations against data from µs-long atomistic MD simulations as well as from experimental SHAPE measurements. Finally, toward ensuring model transferability, we considered the four different types of RNA molecules listed in Table 1 and represented in Figure 1. These systems cover a significant repertoire of different structural elements such as non-canonical base pairs, bulges, junctions and tertiary contacts and were selected with two main criteria, namely: first, they natively adopt a specific fold (i.e. have a stable tertiary structure, which is a prerequisite for ENM applicability) and, secondly, they are amenable to extensive numerical characterization with µs-long MD simulations in explicit solvent. We notice that the size of the studied systems is limited only by the MD computational cost, while the ENM method is straightforwardly applicable to larger molecules, as it has been done for instance in (28).

In the following we discuss the performance of the various models listed in Table 2 starting from those employing a single-bead nucleotide representation and then moving on to the more detailed, multi-bead ones.

Among the one-bead models the best accord with MD data is obtained for the S model, where a nucleotide is represented with the C1′ atom of the sugar moiety. In this case, when the most appropriate elastic interaction range is used (see Table 2), the accord of ENM and MD is significantly larger than the statistical reference (null) case, and not too much behind the accord of the first and second halves of the MD simulations. This result is consistent with the conclusions of the aforementioned recent study of (34) and reinforces them from a significantly broader perspective. In fact, the present assessment is carried out for a wider range of RNA motifs and the search of the optimal representative atom is not limited to the RNA backbone but encompasses the base too.

In this regard, we note that the model with a single bead on the C2 atom of the base (B model) reproduces structural fluctuations less accurately than the S model and the optimal interaction cutoff is more dependent on the specific molecule, a fact that impairs the transferability of the model. These shortcomings are even more evident in the P model, where a nucleotide is represented with the sole phosphorous atom. In fact, both the S and B models are better performing than the P one. The result may be, at first, surprising because of the apparent analogy between the phosphate representation in RNA and the C_α_ representation in proteins. The latter is virtually used in all single-beads ENMs for proteins. However, one should keep in mind a fundamental distinction of backbone and side-group roles for the structural organization and stability of these two types of biopolymers. In fact, whereas for proteins the backbone self-interaction (e.g. hydrogen bonding) contributes significantly to the structural stability, for RNAs the analogous role is, in fact, played by the bases and not by the phosphate groups (78,79). In this regard, it is interesting to recall that RNAs have, in fact, been interpreted as adopting an ‘inside-out’ organization compared to proteins (80). This distinction might help rationalize why the P representation does not serve for RNA ENMs equally well as the C_α_ representation for proteins.

Moving on to two-beads models, we observe that ENMs employing beads both in the bases and in the backbone (SB, BP) perform systematically better than any single-bead model with only a modest increase in the computational complexity. SB and BP models also outperform the SP model. We also stress that being able to reproduce the fluctuations of the bases is by itself an advantage because their functional role is of primary importance in nucleic acids and their dynamics can affect different aspects of the behavior of RNA molecules (see, e.g. (3,70,79)).

Increasing the number of beads featured in the ENM models (see also Supplementary Figure S1 for 5/6-beads model) improves the agreement with MD, consistently with what had been observed for proteins (81). The best overall accuracy is indeed observed for the AA ENM. We focused our attention on this model, as well as on the the SBP model, that uses one bead for each of the sugar, base and phosphate groups. In fact, the consistency of both models with MD data is practically as high as the internal consistency of MD itself. We also note that the optimal performance of the SBP model is attained when the interaction cutoff distance is about equal to 9 Å. This is a convenient feature, as this interaction range falls in the same viable interaction range of elastic networks for proteins (22,25). Furthermore, the typical density of beads in protein ENM is very similar to the SBP model (Table 2). In principle, this allows for the perspective of integration of proteins and RNA elastic networks to study protein/RNA complexes.

The viability of the SBP and AA models is independently underscored by the comparison against experimental SHAPE data, which are notoriously challenging to predict. The challenge is at least partly due to the difficulties of identifying from *a priori* considerations structural or dynamical observables that correlate significantly with SHAPE data. As a first step of the analysis we therefore considered various observables computed from atomistic MD simulations against SHAPE data, and established that the relative fluctuations of consecutive nucleobases provide a viable proxy for SHAPE data. Our comparative analysis showed that such fluctuations can be captured well using the SBP ENM, and to an even better extent with the AA ENM. Possibly, this is a step in the direction of defining a model able to directly correlate three-dimensional structures with SHAPE reactivities. Interestingly, both the ENMs are completely independent from the dihedral potentials and thus should not be directly affected by the pucker conformation of the ribose. The fact that they can provide a reasonable estimate of the backbone flexibility as measured by SHAPE reactivity suggests that the backbone flexibility is mostly hindered by the mobility of the bases.

In conclusion, ENMs were here compared systematically with fully atomistic MD simulations and with SHAPE reactivities. We found that, in spite of their simplistic nature, the three-center (SBP) and AA elastic networks are capable of properly reproducing both MD fluctuations and chemical probing experimental data. Of these two accurate ENMs, the three-center model (SBP) provides an ideal compromise between accuracy and computational complexity, given that retaining the full atomistic detail when modeling large structures, such as the ribosome and other macromolecular RNA/protein complexes, can be computationally very demanding.

A module that implements the ENM for RNA discussed in this paper has been included in the baRNAba analysis tool (http://github.com/srnas/barnaba).

## SUPPLEMENTARY DATA

Supplementary Data are available at NAR Online.

SUPPLEMENTARY DATA
