# Supplementary material for: Elastic network models for RNA: a comparative assessment with molecular dynamics and SHAPE experiments
Source: Nucleic Acids Res. 2015 Jul 17;43(15):7260–9. doi: 10.1093/nar/gkv708 (PMC4551938; doi:10.1093/nar/gkv708)
Supplement: SUPPLEMENTARY DATA [file supp_43_15_7260__index.html]

Elastic network models for RNA: a comparative assessment with molecular dynamics and SHAPE experiments — SUPPLEMENTARY DATA 

# Elastic network models for RNA: a comparative assessment with molecular dynamics and SHAPE experiments

## SUPPLEMENTARY DATA

- SUPPLEMENTARY DATA
