# Supplementary material for: Elastic network models for RNA: a comparative assessment with molecular dynamics and SHAPE experiments
Source: Nucleic Acids Res. 2015 Jul 17;43(15):7260–9. doi: 10.1093/nar/gkv708 (PMC4551938; doi:10.1093/nar/gkv708)
Supplement: SUPPLEMENTARY DATA [file supp_gkv708_nar-01021-r-2015-File005.pdf]

Elastic network models for RNA: a comparative assessment with molecular dynamics and SHAPE experiments

G. Pinamonti, S. Bottaro, C. Micheletti, G. Bussi

Supplementary Data

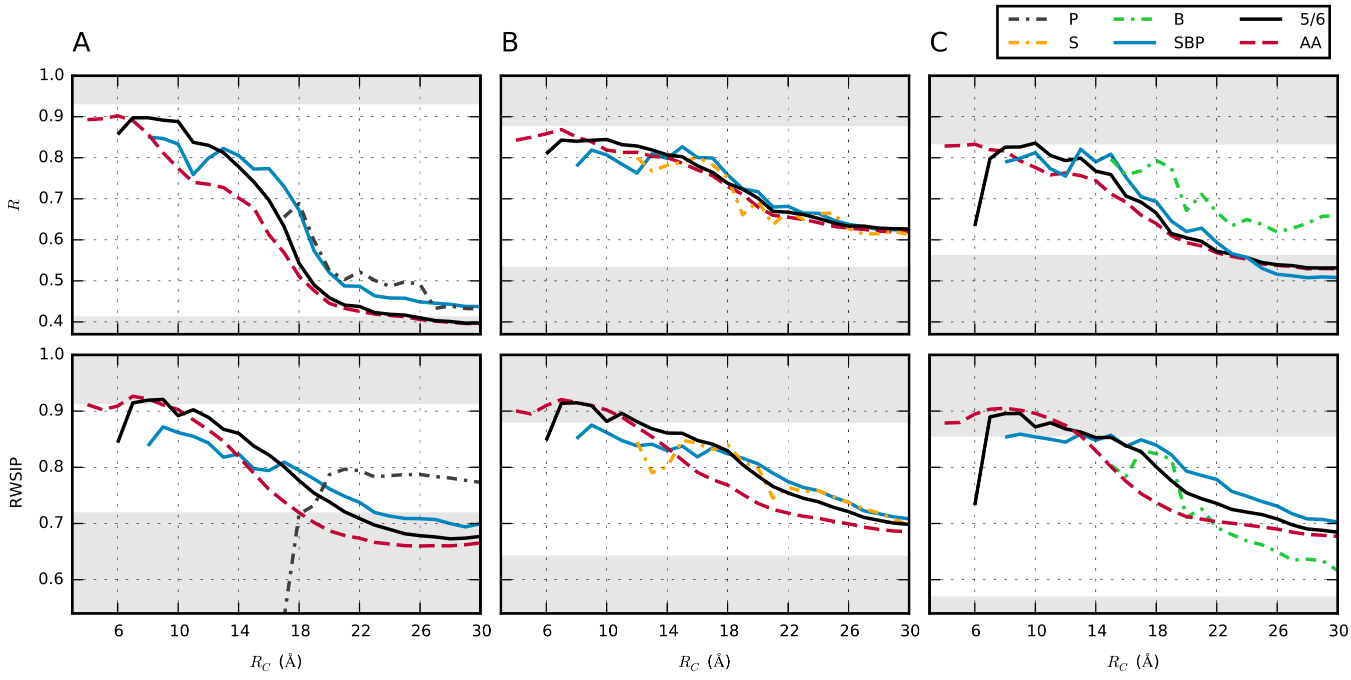

Figure SD 1: Comparison between the MD simulations and a ENM constructed considering 5 or 6 beads per nucleotide: P, C1′, C4′, C2, C5 for pyrimidines; the same plus C8 for purines. Also the 1-,3-beads models and the all-atom model are shown for comparison. Fluctuations' correlation (upper panels) and RWSIP (lower panels) are computed considering the beads in the base (A), sugar (B) and phosphate (C), and are averaged over the four molecules considered.

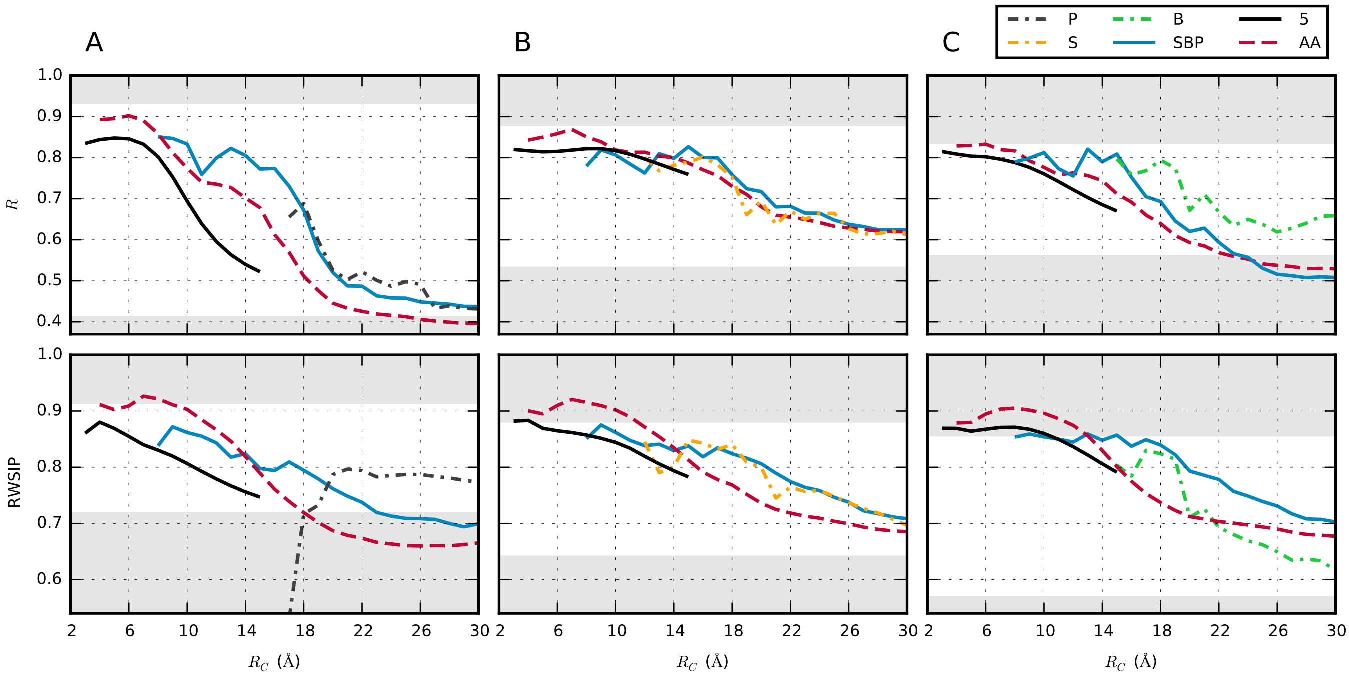

Figure SD 2: Comparison between the MD simulations and a 3-beads SBP ENM with space-dependent elastic constant. The elastic constant is given by $k_{ij} = \exp(-(d_{ij}/R_C)^2)$. The 3-beads SBP model with sharp cutoff is shown for comparison. Fluctuations' correlation (upper panels) and RWSIP (lower panels) are computed considering the beads in the base (A), sugar (B) and phosphate (C), and are averaged over the four molecules considered.

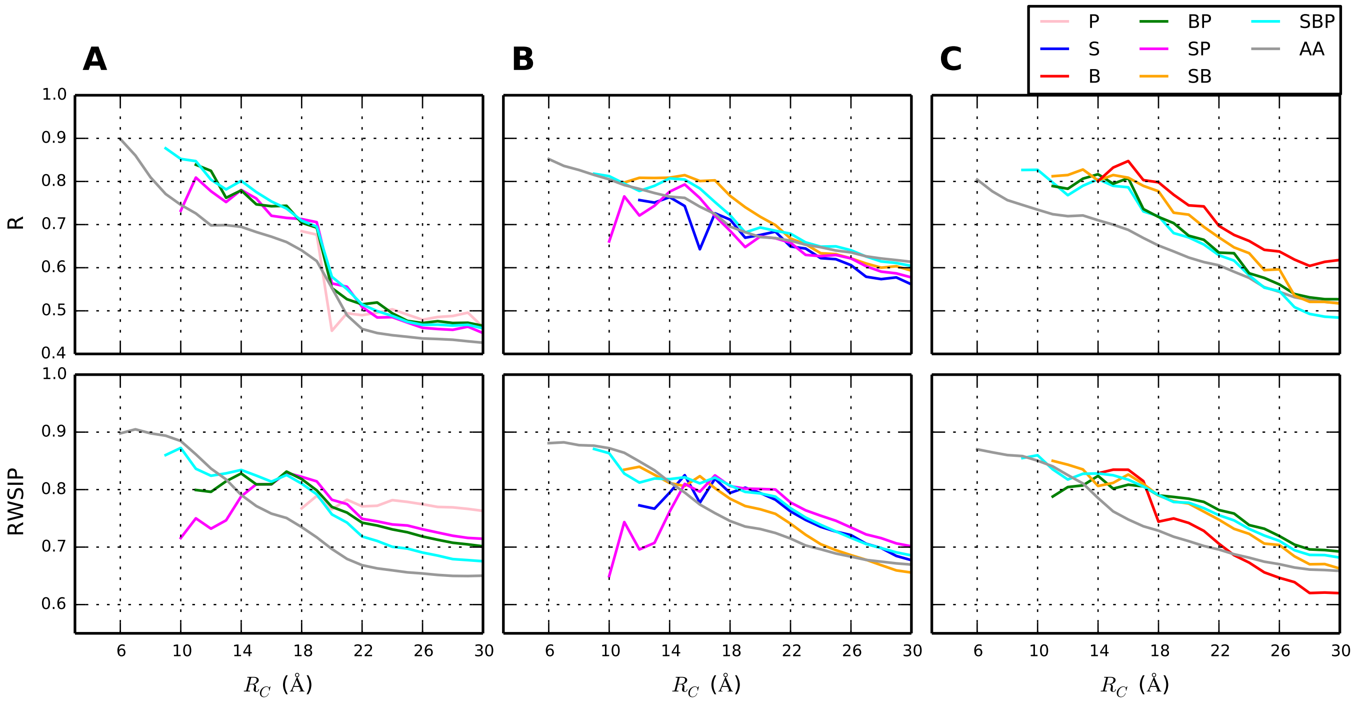

Figure SD 3: Comparison between the MD simulations and the ENMs using as references the experimental structures. Fluctuations' correlation (upper panels) and RWSIP (lower panels) are computed considering the beads in the base (A), sugar (B) and phosphate (C), and are averaged over the four molecules considered.

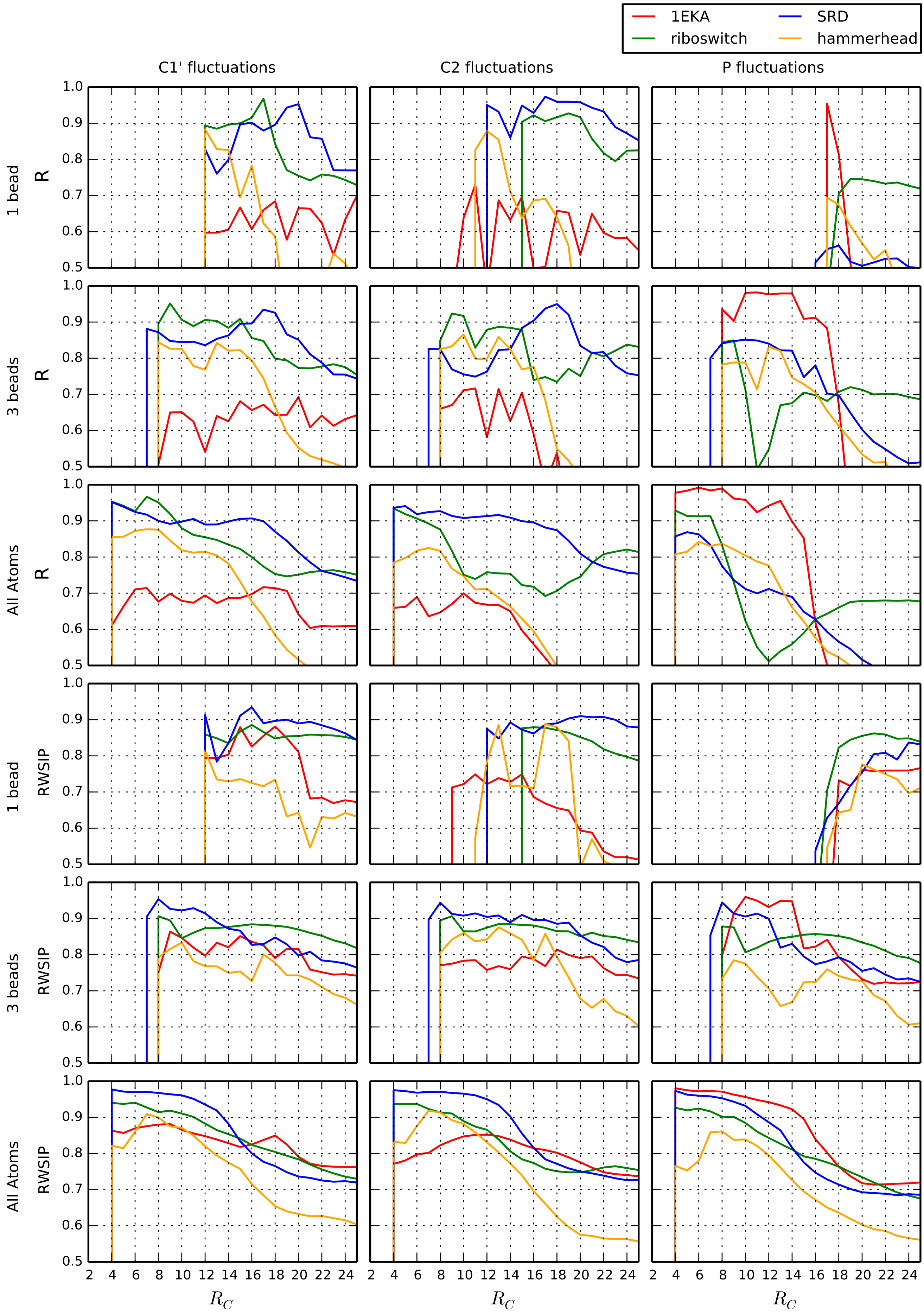

Figure SD 4: Agreement between MD simulations and ENM for different radii of cutoff. Correlation between MSF (upper panels), and RWSIP (lower panels). The results are shown separately for the 4 different molecules for the 1 bead, 3-beads SBP and for the all atom model, as labeled. Left: phosphate beads; middle: sugar beads; right: nucleobase beads.

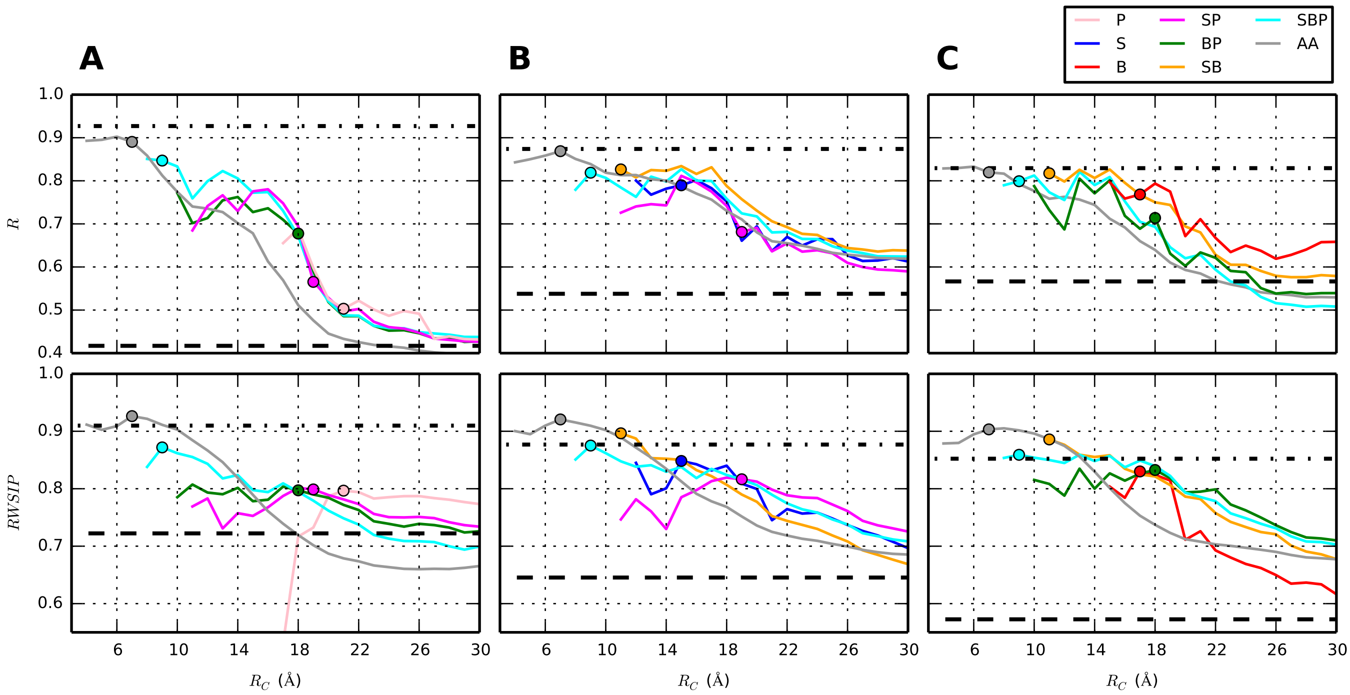

Figure SD 5: Agreement between MD and ENM. Comparison of all the considered models with 1,2,3 beads per nucleotide as well as the all-atoms model. Values at the optimal cutoff values are represented by circles. Fluctuations' correlation (up) and RWSIP (down) are computed considering the beads in base (A), sugar (B) and phosphate (C), and are averaged over the four molecules studied.

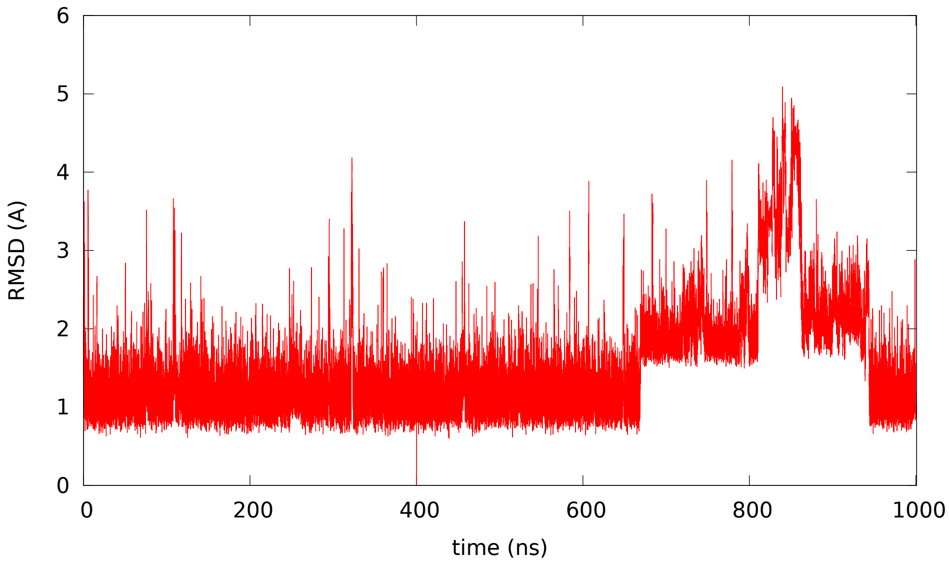

Figure SD 6: RMSD of the duplex along the 1 μs trajectory, computed with respect of the centroid frame. The increase in the RMSD visible at $t \simeq 670$ ns correspond to a base fraying event.

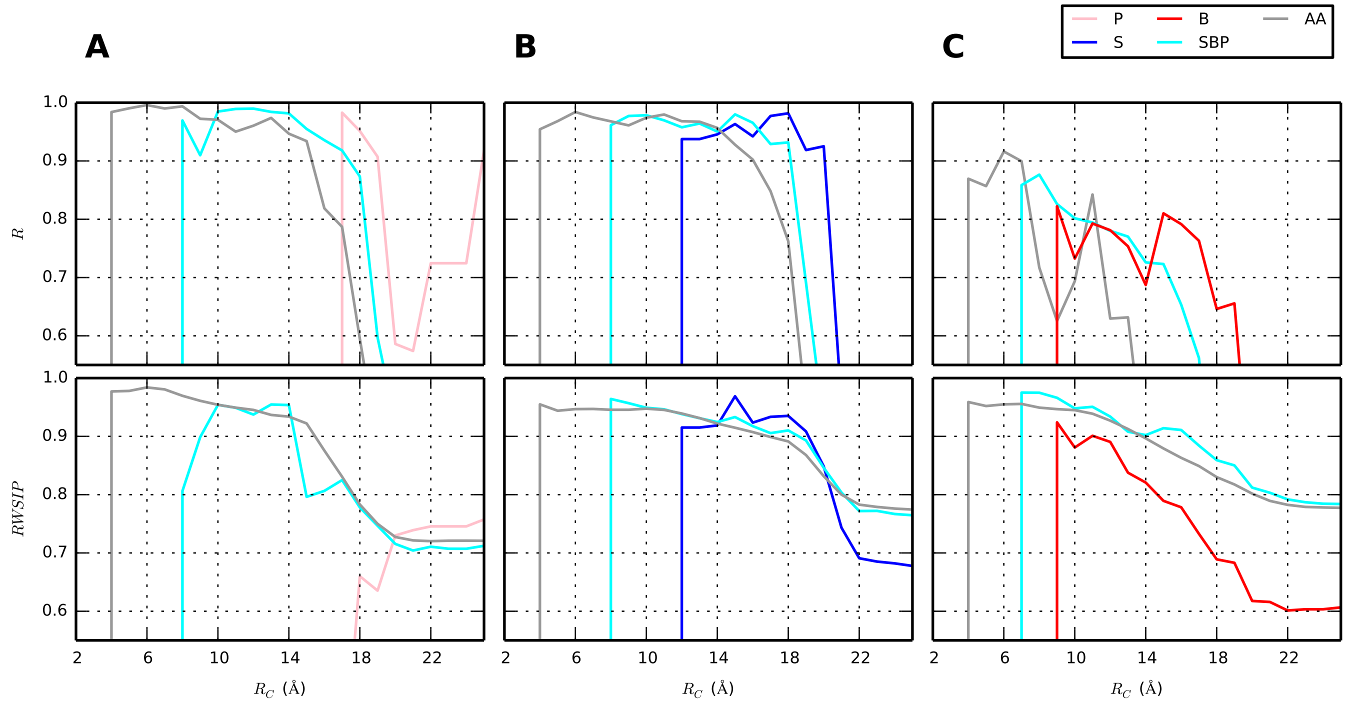

Figure SD 7: Agreement between MD and ENM on the duplex molecule excluding the terminal residues from the analysis. Fluctuations' correlation (up) and RWSIP (down) are computed considering the beads in base (A), sugar (B) and phosphate (C).

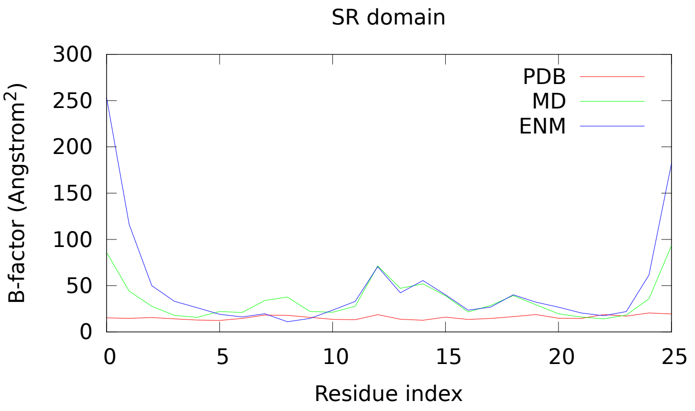

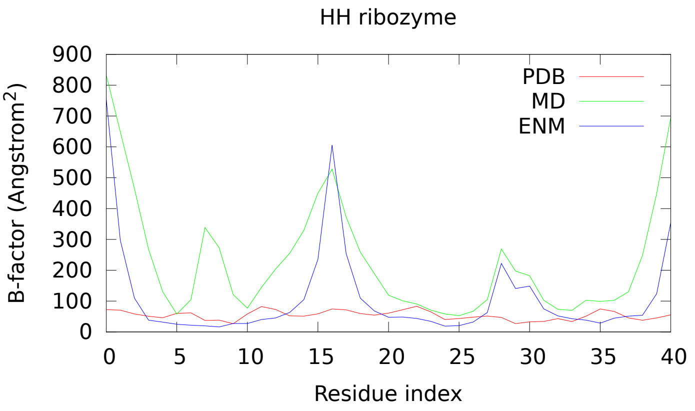

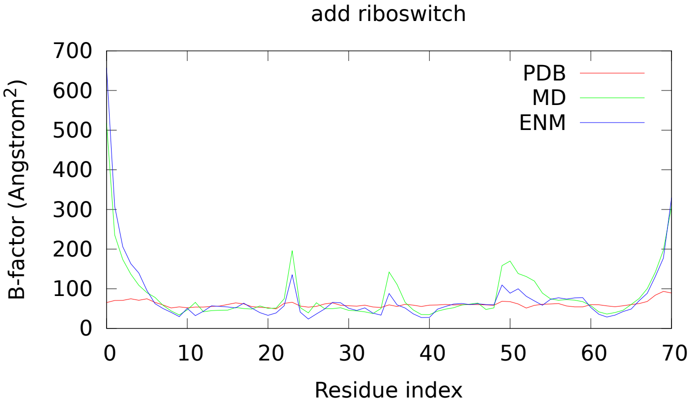

Pearson correlation coefficients

Molecule	PDB-MD	PDB-ENM	MD-ENM
riboswitch	0.57	0.52	0.95
SR domain	0.31	0.17	0.88
HH ribozyme	0.22	0.30	0.83

Figure SD 8: Comparison between the B-factors relative to the C1′ atoms, predicted by ENM, computed from MD simulations and extracted from the PDB files. The correlation between the experimental B-factors and the values predicted by ENM and MD are significantly lower than the correlation between ENM and MD. This can be explained considering that the experimental B-factors include many effects, such as crystal contacts and lattice defects, and thus are not guaranteed to provide a reliable account of the local amplitude of motion for a molecule in solution.

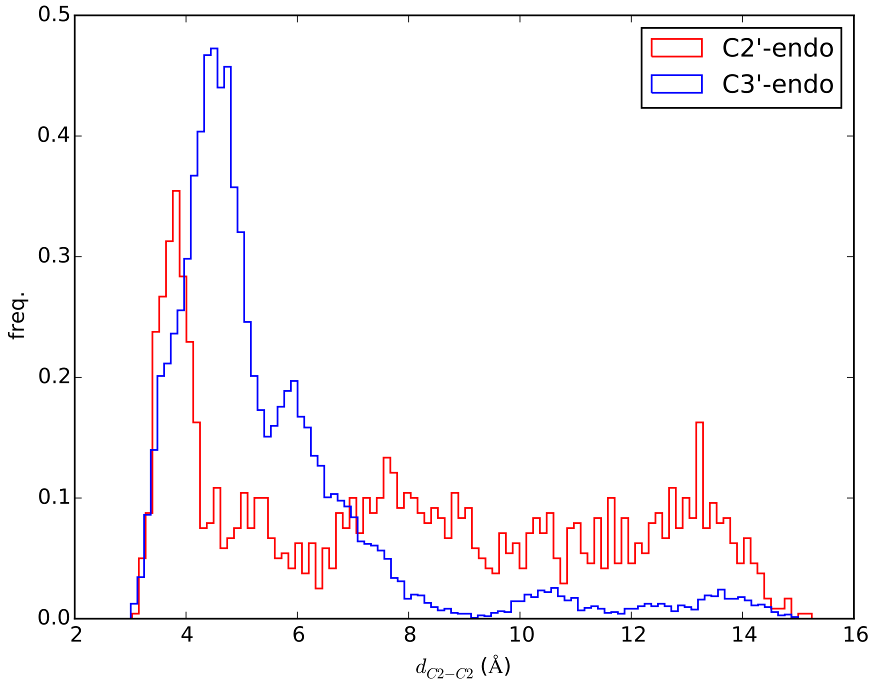

Figure SD 9: Distribution of the C2-C2 distances recorded during the MD simulation of the *add* riboswitch for residues in C2′-endo/ C3′ conformation. The pucker conformation was determined from the pseudorotation phase P, computed using the baRNAba analysis tool (`http://github.com/srnas/barnaba`). We identified C2′-endo conformation with values of P between 100 and 250 degrees.

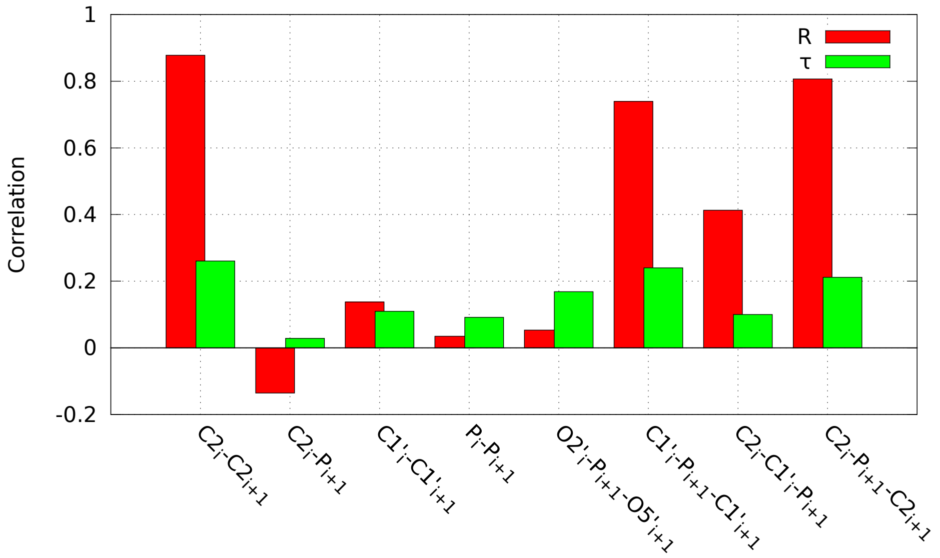

Figure SD 10: Comparison between SHAPE reactivities and distance/angle fluctuations, computed from an atomistic MD simulation. The SHAPE reactivities relative to the i-th residues are compared with the fluctuations between atoms belonging to both the considered residue and the one following in the chain. The two profiles are then compared by mean of the Pearson linear correlation coefficient (R) and the Kendall rank correlation coefficient (τ).

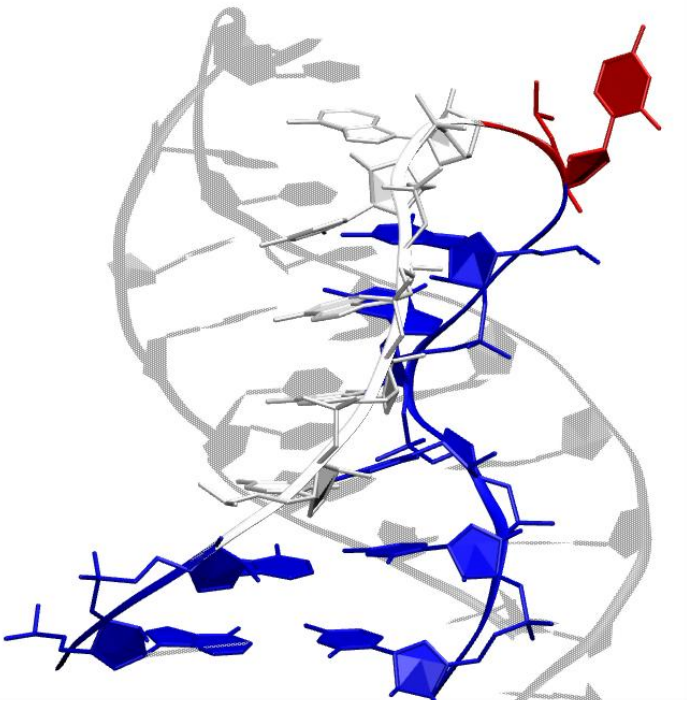

Figure SD 11: Example of nucleotides that do not form pairing interaction and have either low or high SHAPE reactivity. Residues 31 to 35 from *add* riboswitch, enlighted in white, have low SHAPE reactivity (< 0.5); they do not form Watson-Crick or Wobble pairs, but they are stabilized by stacking interactions. Residue 36, in red, presents an high reactivity ($\simeq 2.07$).
